# Cost-effectiveness of rotavirus vaccination in Mozambique

**DOI:** 10.1016/j.vaccine.2022.07.044

**Published:** 2022-08-26

**Authors:** Esperança Lourenço Guimarães, Assucênio Chissaque, Clint Pecenka, Andrew Clark, Basília Vaz, Arlindo Banze, Neide Canana, Clésio Romão, Maria do Rosário Oliveira Martins, Nilsa de Deus, Frédéric Debellut

**Affiliations:** a Instituto Nacional de Saúde, Marracuene district, EN1, Bairro da Vila – Parcela no 3943, Maputo, Mozambique; b Instituto de Higiene e Medicina Tropical (IHMT), Universidade Nova de Lisboa, Rua da Junqueira 100, 1349-008 Lisbon, Portugal; c Center for Vaccine Innovation and Access, PATH, Seattle, WA, United States; d Department of Health Services Research and Policy, London School of Hygiene & Tropical Medicine, London, United Kingdom; e Ministry of Health, Maputo, Mozambique; f Malaria Consortium, Maputo, Mozambique; g Departamento de Ciências Biológicas, Universidade Eduardo Mondlane, Maputo, Mozambique; h Center for Vaccine Innovation and Access, PATH, Geneva, Switzerland

**Keywords:** Diarrhea, Rotavirus, Vaccination, Modelling, UNIVAC, Cost-effectiveness, Mozambique

## Abstract

**Introduction:**

Rotavirus is one of the most common cause of severe gastroenteritis in children, with the largest mortality burden in low- and middle-income countries. To prevent rotavirus gastroenteritis, Mozambique introduced ROTARIX® vaccine in 2015, however, its cost-effectiveness has never been established in the country. In 2018, additional vaccines became available globally. This study estimates the cost-effectiveness of the recently introduced ROTARIX in Mozambique and compares the cost-effectiveness of ROTARIX®, ROTAVAC®, and ROTASIIL® to inform future use.

**Methods:**

We used a decision-support model to calculate the potential cost-effectiveness of vaccination with ROTARIX compared to no vaccination over a five-year period (2016–2020) and to compare the cost-effectiveness of ROTARIX, ROTAVAC, and ROTASIIL to no vaccination and to each other over a ten-year period (2021–2030). The primary outcome was the incremental cost per disability-adjusted life-year (DALY) averted from a government perspective. We assessed uncertainty through sensitivity analyses.

**Results:**

From 2016 to 2020, we estimate the vaccine program with ROTARIX cost US$12.3 million, prevented 4,628 deaths, and averted US$3.1 million in healthcare costs. The cost per DALY averted was US$70. From 2021 to 2030, we estimate all three vaccines could prevent 9,000 deaths and avert US$7.8 million in healthcare costs. With Global Alliance for Vaccines and Immunization (Gavi) support, ROTARIX would have the lowest vaccine program cost (US$31 million) and 98 % probability of being cost-effective at a willingness-to-pay threshold of 0.5x GDP per capita. Without Gavi support, ROTASIIL would have the lowest vaccine program cost (US$75.8 million) and 30 % probability of being cost-effective at the same threshold.

**Conclusion:**

ROTARIX vaccination had a substantial public health impact in Mozambique between 2016 and 2020. ROTARIX is currently estimated to be the most cost-effective product, but the choice of vaccine should be re-evaluated as more evidence emerges on the price, incremental delivery cost, wastage, and impact associated with each of the different rotavirus vaccines.

## 1 Introduction

Globally, diarrhea is the third leading cause of mortality in children under five years of age [1]. Rotavirus (RV) is the most common cause of severe diarrhea in young children worldwide, with most hospitalizations and deaths occurring in low- and middle-income countries (LMICs) [2], [3]. Despite efforts to reduce the global burden of RV, in 2019 it was responsible for approximately 150,000 deaths among children under five years of age, most of them (81 %) in sub-Saharan Africa [4]. Prior to the introduction of ROTARIX® (GlaxoSmithKline (GSK) Biologicals, Rixensart, Belgium) in 2015, the annual mortality rate in Mozambique was estimated to be around 48 (19 – 90) per 100,000 children under five years of age [5].

Vaccination is one of the most effective ways to prevent RV gastroenteritis (RVGE). There are currently four RV vaccines prequalified by the World Health Organization (WHO) for global use: the pentavalent RotaTeq® (Merck & Co., USA), the monovalent ROTARIX, the pentavalent ROTASIIL® (Serum Institute of India Pvt. Ltd. India), and the monovalent ROTAVAC® (Bharat Biotech, India) [6]. These vaccines have been reported to be effective in numerous countries in reducing the number of RV cases, hospitalizations, and deaths [7], [8], [9], [10].

Mozambique introduced ROTARIX in September 2015 through the Expanded Program on Immunization (EPI) as a strategy to reduce the burden of RV infections and hospitalizations. The vaccine has already had a positive impact on gastroenteritis hospital admissions in children < 5 years of age, showing a reduction in the RV-positive proportion from 40.5 % in pre-vaccine period to 13.5 % in post-vaccine period [11].

As of October 2021, RV vaccines have been introduced in 110 countries [12]. Several studies have shown that RV vaccination is a cost-effective intervention for prevention of severe diarrhea, especially in countries with a high child mortality rate [13], [14], [15], [16], [17], [18]. However, there are no known published data on the impact and cost-effectiveness of ROTARIX or other available RV vaccines in Mozambique. The country currently benefits from financial support from Gavi, the Vaccine Alliance (Gavi). However, as the economic situation of the country improves, this support will gradually decrease to the point where the government will have to fully self-finance vaccine costs [19]. From 2018 to 2020 Mozambique had an average Gross National Income (GNI) per capita of US$470 [20]. Upon reaching the eligibility threshold (average GNI per capita of US$1630 over a three-year period), the country will begin a five-year transition towards full self-financing [21]. Cost-effectiveness analyses can provide important evidence to decision-makers about the health and cost consequences of the current use of ROTARIX, both in the context of financial support from Gavi and in the absence of such support. It can also be used to compare ROTARIX to alternative RV vaccines (e.g., ROTAVAC and ROTASIIL) with different product characteristics. This should help to support national strategic planning and priority setting in the context of a constrained budget for public health interventions.

This study aims to assess the health and economic impact of the recently introduced ROTARIX into Mozambique’s EPI and to calculate and compare the cost-effectiveness of three products (ROTARIX, ROTAVAC, and ROTASIIL) that could be used in future.

## 2 Methods

### 2.1 Study design and model

For this analysis we used the universal vaccine cost-effectiveness and impact modelling framework (UNIVAC) proportionate outcomes decision-support model (version 1.4.16). This was developed in Microsoft Excel (Excel, Microsoft Corp, Redmond, WA, US) to allow transparent assessment of the cost-effectiveness of different vaccines, including RV vaccines [22]. The model has a user-friendly interface and was specifically designed for use by national multidisciplinary teams in LMICs [23].

We ran two separate analyses. In the first, the cost-effectiveness of ROTARIX was compared to no vaccination over the five-year period 2016–2020. In the second, we calculated the cost-effectiveness of ROTARIX, ROTAVAC, and ROTASIIL compared to no vaccination, and to each other, over the ten-year period 2021–2030. We also estimated the cost-effectiveness of the three vaccines as if they had the same health impact. ROTATEQ was not included in this analysis because it is not available for Gavi-supported countries [6].

For each birth cohort included in the evaluation, RV cases, visits, hospital admissions, deaths, vaccine program costs, and healthcare costs were calculated over the first five years of life. Disability-adjusted life-years (DALYs) were calculated over the lifetimes of all cohorts evaluated. DALYs account for both years lost due to early death and years lived with the disease, which facilitates comparison with other potential public health interventions [24].

The primary outcome measure was the cost (US$) per DALY averted [24]. We used 2018 US$ (United States Dollars) for all costs. Both future health outcomes and costs were discounted at 3 % to reflect the time preference for immediate benefits and the opportunity of investing present capital, as recommended by WHO [25]. All results were calculated from a government healthcare cost perspective. This excludes any costs borne by households when seeking treatment at public or private health care providers, e.g., out-of-pocket medical expenses, travel costs, lost earnings of caregivers, etc. The government perspective also excludes the sizable contribution paid by Gavi to the EPI. A separate ‘what-if’ scenario was evaluated to calculate the cost-effectiveness assuming the government was fully self-financing the program.

A willingness-to-pay (WTP) threshold, which is a value used to represent “an estimate of what an individual might be prepared to pay for one year lived healthily,” allows cost–effectiveness ratios (US$ per DALY averted) to be interpreted [26]. Mozambique has not yet defined a country-specific WTP threshold, thus we assumed a threshold of 0.5 times (x) GDP per capita [15], [27]. However, we produced outputs that would allow interpretation of our results at different WTP thresholds.

### 2.2 Data collection and consensus building

In 2016, the population of Mozambique was 27,829,930 [28]. Demographic projections for cohorts born in the period 2016–2030 were obtained from United Nations Population (UNPOP) projections and included an average population size of 990,221 (cohort from 2016 to 2020) and 1,094,020 (cohort from 2016 to 2021)by age/year, life-expectancy by age/year, and under-five mortality rates by year [29]. For all other parameters (e.g., RVGE disease burden, vaccine coverage, timeliness, efficacy, use and costs of health services, and RV vaccination program costs), a national multidisciplinary working group on RV was convened to identify and agree on the most appropriate data (and uncertainty ranges) to populate the model. This working group was convened to provide updated evidence to the National Immunization Technical Advisory Group (NITAG), known in Mozambique as *Comité de Peritos de Imunização* (CoPI), whose role is to make health policy and strategic decisions based on scientific evidence. The RV working group was composed of members from the Ministry of Health (MoH) of Mozambique namely the EPI, experts in RV diarrheal disease from the National Institute of Health (*Instituto Nacional de Saúde* – INS), and members from non-governmental organisations such as United Nations Children's Fund, John Snow Inc., WHO, and Village Reach. The group met four times in 2021 (July, Aug, Sept, Dec) to build consensus on the input parameters and scenarios included in the model.

### 2.3 Disease burden

To estimate the incidence of severe symptomatic RVGE cases (per 100,000 per year, aged < 5 years), we combined regional estimates of the rate of all-cause severe gastroenteritis with the mean RV-positive proportion in Mozambique, as estimated by three international sources, namely the Global Burden of Disease (GBD) study, WHO, Centre for Disease Control, and the Maternal and Child Epidemiology Estimation Group [15]. The definition of the severity of diarrhea is based on Vesikari Score which was developed to help access the effectiveness and efficacy of rotavirus vaccine on 20 points which allows combine different symptoms such as diarrhea and vomit episodes, dehydration status, type of treatment and others [30]. The incidence of non-severe RVGE cases was then calculated by subtracting the incidence of severe RVGE cases from the incidence of any symptomatic RVGE cases, obtained from a systematic review and meta-analysis of LMICs from the African region [31]. The rate of RVGE outpatient visits was taken from a modelling study by Debellut et al [15]. To estimate the rate of RVGE hospital admissions, we calculated the number of hospital admissions due to diarrhea in children aged < 5 years based on data from Horn et al [32] and Farthing et al [33] and then multiplied this by the RV-positive proportion (38.5 %) for Mozambique [11]. We assumed that only severe cases would progress to hospital admission. The RV mortality rate (for the pre-vaccination era, i.e., 2015) was obtained from the GBD study [5] and the disability weights were gathered from Salomon et al. [34]. All the disease burden input values are shown in Table 1.

**Table 1:** Input parameters for estimating the burden of diarrhea in Mozambique.

**Parameter**	**Central value**	**Scenarios**		**Source**
		**Lower bound**	**Higher bound**	
**Incidence (per 100,000 under-five children)**				
Non severe RVGE cases	7,473	5,224	10,870	[31]
Non severe RVGE visits	685	239	2,489	[15]
Severe RVGE cases	2,527	1,776	3,130	[15]
Severe RVGE visits	2,315	1,627	2,867	[15]
Severe RVGE hospitalizations	807	605	1,009	Adapted based on [11], [32], [33]
Severe RVGE deaths	48	19	90	[1]
**Disability weights**				
Non-severe RVGE	0.19	0.13	0.26	[34]
Severe RVGE	0.25	0.16	0.35	[34]
**Mean duration of illness**				
Non-severe RVGE	5	2	6	Assumption [17]
Severe RVGE	7	5	9	Assumption [17]
**RVGE age distribution**	**Cumulative percentage**			
<1 month	0 %	–	–	Adapted based on [11]
<2 months	1 %	–	–	Adapted based on [11]
<3 months	6 %	–	–	Adapted based on [11]
<6 months	28 %	–	–	Adapted based on [11]
<1 year	70 %	–	–	Adapted based on [11]
<2 years	94 %	–	–	Adapted based on [11]
<3 years	98 %	–	–	Adapted based on [11]
<4 years	99 %	–	–	Adapted based on [11]
<5years	100 %	–	–	Adapted based on [11]

RV disease age distribution data were adapted from a study based on the national diarrhea surveillance in the pre-vaccine period (2014–2015) [11]. A parametric curve (Burr distribution) was fitted to a standard set of age distribution data points to allow more granular estimation of the proportion of RVGE disease occurring in each week of age < 5 years. Methods for age fitting have been described elsewhere [35], [36].

For all parameters where there is perceived uncertainty in the data, we provided a low and high range for sensitivity analyses. If 95 % confidence intervals were not available, we assumed a wide range by subtracting or adding 25 % of the base case input value [37], [38].

### 2.4 Vaccine coverage and timeliness

For the 2016 to 2020 cohorts, coverage of the first and second dose of ROTARIX vaccine was assumed to be 90 % and 88 %, respectively. This was based on the reported coverage of the last dose in 2019 (88 %) and allowing for expected drop-out between the first and second doses [39].

For the cohorts from 2021 to 2030, the coverage of all doses administered within the two-dose (ROTARIX) and three-dose (ROTASIIL and ROTAVAC) RV vaccines was assumed to be the same as diptheria, tetanus, and pertussis (DTP1) (93 %), DTP2 (91 %) and DTP3 (88 %), since these vaccines are provided at the same time. We used 2019 coverage rates of DTP1 and DTP3 [39] and assumed that the average between DTP1 and DTP3 would correspond to DTP2 coverage.

The timeliness (coverage by age) of DTP1, 2, and 3 vaccinations was used as a proxy for the timeliness of the first, second, and third dose of RV vaccines. A gamma curve was fitted to the Demographic and Health Survey (DHS) data for 2015 to allow estimation of timeliness by week of age < 5 years.

### 2.5 Vaccine efficacy

**Fig. 1:**
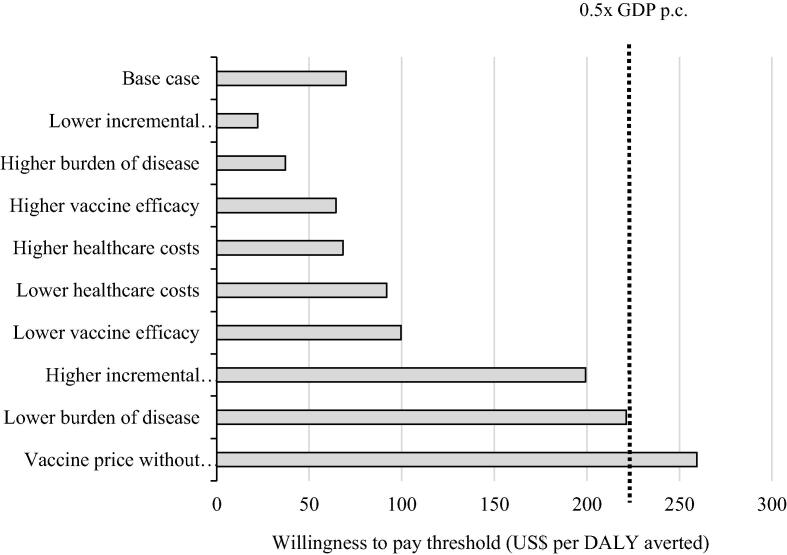
Scenario analysis results, showing incremental cost-effectiveness ratio (US$ per DALY averted) of ROTARIX, compared to no vaccination.

In the absence of head-to-head data from the same trial population, we assumed equivalent vaccine efficacy and waning for all RV vaccines. According to a meta-regression of randomised controlled trials, efficacy two weeks after the first dose is 49.9 % (38.2–65.3 %) and efficacy two weeks after the final dose is 78.9 % (75.5–82.3 %). This analysis calculated the efficacy of live oral RV vaccines in countries with high under-five mortality, including Mozambique. Substantial declines in vaccine protection over time were also assumed, based on the same analysis [40]. We assumed the same level of efficacy and the same rate of waning protection after the second and third dose. This assumption therefore favoured the vaccines with three doses as this schedule delays the onset of waning protection. However, due to substantial uncertainty about this assumption, we also showed the results with the assumption of equal overall impact irrespective of the vaccine product used (Supplementary file I - Fig. 1). Since UNIVAC is a static proportionate outcomes model, any herd effect of the vaccine was not considered in the analysis.

### 2.6 Vaccination cost

Because Mozambique is eligible for vaccine financial support from Gavi, the government only co-finances part of the vaccine cost, which is currently US$0.40 per course for any vaccine [21]. This value has been used in the model for the base-case scenario and is assumed to be fixed over both periods evaluated (2016–2020 and 2021–2030). However, the full per-course price of the vaccines (US$ 4.66 for ROTARIX, US$ 3.42 for ROTAVAC, and US$ 2.85 for ROTASIIL), assuming no support from Gavi, was used for scenario analysis [6], again assuming the price would be fixed over the entire period of the analysis.

The EPI team chose to analyse ROTASIIL in its two-dose vial, lyophilised presentation (US$0.95 per dose), ROTAVAC in its five-dose vial, liquid presentation (US$1.14 per dose), and ROTARIX in its one-dose vial, liquid presentation (US$ 2.33 per dose) after careful consideration of the price per dose, wastage, volume, and storage conditions.

The vaccination cost per child was calculated based on the vaccine price, wastage [6], international handling (procurement process) [41], international delivery (transportation), and immunization delivery cost (Table 2). The immunization delivery cost is the additional cost to the health system that would be involved in adding the vaccine to the current vaccine delivery system and represents expenses related to supply chain, capital, labour, and other service delivery to implement the vaccination in the country [42].

**Table 2:** Input parameters for estimating ROTARIX, ROTAVAC, and ROTASIIL program costs.

**Parameter**	**Central value**	**Scenarios**		**Source**
		**Lower bound**	**Higher bound**	
**Vaccine price per dose (US$) – with Gavi support**				
ROTARIX	0.20	–	–	[6]
ROTASIIL	0.13	–	–	[6]
ROTAVAC	0.13	–	–	[6]
**Vaccine price per dose (US$) – without Gavi support**				
ROTARIX	2.33	–	–	[6]
ROTASIIL	0.95	–	–	[6]
ROTAVAC	1.14	–	–	[6]
**OTHER COSTS**				
**Wastage rate (% of vaccine)**				
ROTARIX	4.00 %	2.00 %	6.00 %	[6]
ROTASIIL	9.00 %	7.50 %	9.40 %	[6]
ROTAVAC	13.00 %	7.50 %	9.40 %	[6]
International handling (all vaccines)	3.00 %	1.40 %	4.50 %	[41]
International delivery (all vaccines)	6.00 %	2.00 %	15.00 %	[41]
Safety box/bag per dose (US$) - all vaccines	0.02	0.02	0.03	[47]
Incremental delivery cost per dose (US$) - all vaccines	1.17	0.39	2.78	[42]

### 2.7 Healthcare costs

Country-specific estimates of healthcare treatment costs borne by the government for clinic visits and hospital admissions were obtained from a systematic review of literature published between 2006 and 2018 on the cost of childhood diarrhea across 137 LMICs [43] (Table 3).

**Table 3:** Input parameters for estimating health service costs (2018 US$).

**Parameter**	**Central value**	**Scenarios**		**Source**
		**Lower bound**	**Higher bound**	
**Non-severe RVGE**				
Government cost of RVGE outpatient visit (US$)	4.47	2.23	6.70	[43]
**Severe RVGE**				
Government cost of RVGE outpatient visit (US$)	4.47	2.23	6.70	[43]
Government cost of RVGE hospitalization (US$)	19.62	9.81	29.44	[43]

### 2.8 Deterministic and probabilistic sensitivity analysis

To assess the impact of uncertainties introduced by each parameter provided in Table 1, Table 2, Table 3, one-way sensitivity analysis was performed to understand the variation of the cost-effectiveness results in scenarios that are less or more favourable to the vaccine [24], [25]. For **less favourable scenarios,** we considered: upper bound of incremental delivery cost per dose, vaccine price without Gavi support, lower bound of disease burden parameters, lower bound of vaccine efficacy, and lower bound of healthcare cost. For **more favourable scenarios,** we considered: upper bound of disease burden parameters, upper bound of vaccine efficacy, lower bound of incremental delivery cost per dose, and upper bound of healthcare costs. We also looked at ROTARIX cost-effectiveness with equivalent impact of three-dose vaccines, and at ROTAVAC and ROTASIIL with equivalent impact of two-dose vaccines, to assess how the number of doses impacts the cost per DALY averted. Furthermore, a probabilistic sensitivity analysis (PSA) was performed by varying all parameters simultaneously within their ranges, with 1,000 iterations of a Monte Carlo simulation to yield a range of possible values for costs and outcomes. For simplicity, a transparent Beta-PERT distribution was assumed for all parameters and their ranges. The proportion of probabilistic runs with ICERs below different WTP thresholds reflected the probability that RV vaccination would be cost-effectiveness at these thresholds.

## 3 Results

### 3.1 Cost-effectiveness of ROTARIX from 2016 to 2020

Under the **base-case scenario**, from 2016 to 2020, we estimated that use of ROTARIX in Mozambique prevented 963,701 RVGE cases, including 269,784 severe cases (42 % reduction) and 4,628 deaths (42 % reduction). This corresponds to 286,178 discounted DALYs averted and around US$3.1 million avoided (45 % reduction) in RVGE treatment costs from the government perspective (Table 4).

**Table 4:** Projected impact and cost-effectiveness of RV vaccination in cohorts vaccinated over the period 2016–2020 and 2021–2030 (DALYs discounted), government perspective.

	**2016**–**2020**	**2021**–**2030**		
	**ROTARIX**	**ROTARIX**	**ROTAVAC**	**ROTASIIL**
**HEALTH OUTCOMES**				
Non-severe cases averted	693,917	1,568,970	1,800,582	1,800,582
Severe cases averted	269,784	624,120	700,037	700,037
Outpatients’ visits averted	321,253	715,515	833,589	833,589
Hospitalizations averted	86,972	199,326	225,676	225,676
Deaths averted	4,628	8,067	9,198	9,198
DALYs averted	286,178	522,905	595,410	595,410
**ECONOMIC OUTCOMES**				
**Health treatment costs averted (US$)**				
Healthcare treatment costs	3,113,296	7,106,570	8,078,406	8,078,406
**Vaccination programme cost (US$)**				
With Gavi support	12,251,605	31,030,830	40,791,230	40,449,222
Without Gavi support	35,395,396	84,744,515	85,318,192	75,785,017
**Cost per DALY averted (compared to no vaccine) (US$)**				
With Gavi support	70	102	122	121
Without Gavi support	–	330	295	259
Proportion of the GDP per capita (US$448)	16 %	23 %	27 %	27 %
**Cost per DALY averted compared to ROTARIX (US$) (with Gavi support)**				
ROTAVAC compared to ROTARIX	–	–	20	–
ROTASIIL compared to ROTARIX	–	–	–	19

The cost of vaccine implementation with Gavi support was projected to be around US$12.3 million over the 5-year period, representing an average of US$2.5 million annually. However, it was partially balanced by the health care costs averted. Annually, an average of US$622,659 in treatment costs was averted from the government perspective (42 % reduction).

We calculated a cost of US$70 per DALY averted (95 % UI, 36–159) for ROTARIX vaccination, from the government perspective, compared to no vaccination. This was below the WTP threshold of 0.5x the national GDP per capita. Further, scenario analysis showed that the cost-effectiveness was below this threshold in most scenarios. ROTARIX was not below the WTP threshold in the scenario of vaccine price without Gavi support (US$259 per DALY averted) (Fig. 1).

### 3.2 Cost-effectiveness of ROTARIX, ROTAVAC, and ROTASIIL from 2021 to 2030

ROTAVAC and ROTASIIL were estimated to prevent more RVGE health outcomes and RVGE treatment costs than ROTARIX (Table 4), because we assumed the same vaccine efficacy after the second and third dose. However, there is substantial uncertainty about this assumption, as explained above. With Gavi support, the vaccine program cost was lowest for ROTARIX (US$31 million) compared to ROTASIIL (US$40.4 million) and ROTAVAC (US$40.8 million). Without Gavi support, the vaccine program cost was lowest for ROTASIIL (US$75.8 million) compared to ROTARIX (US$84.7 million) and ROTAVAC (US$85.3).

**Fig. 2:**
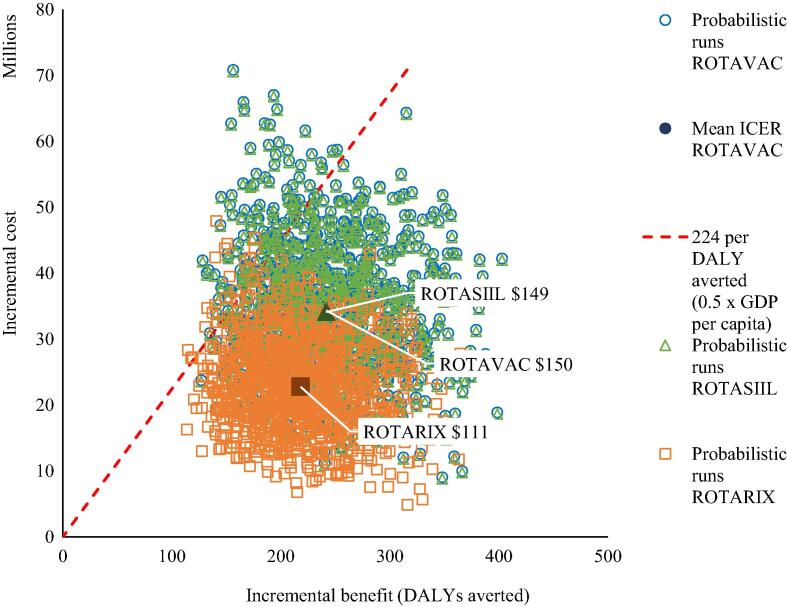
Cost-effectiveness plane showing the incremental costs and benefits of vaccination with ROTARIX, ROTAVAC, and ROTASIIL, compared to no vaccination.

With Gavi support, the cost-effectiveness of the lowest cost product (ROTARIX) was US$102 per DALY averted (95 % UI, 40–221), compared to no vaccination. Both ROTAVAC and ROTASIIL were dominated because they provided similar benefits at greater cost (Fig. 2).

Table 4 also shows that without Gavi support, the cost-effectiveness of the lowest cost product (ROTASIIL) was US$259 per DALY averted (95% UI, 147–466), compared to no vaccination. In this scenario both ROTARIX and ROTAVAC were dominated because they provided similar benefits at greater cost. Additional information on the cost-effectiveness of vaccination from 2021 to 2030 with and without Gavi support is presented in the Supplementary file - Fig. 2.

**Fig. 3:**
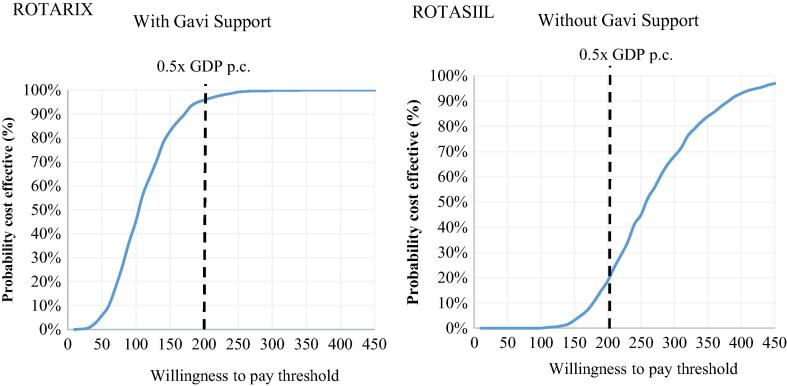
Cost-effectiveness acceptability curve for the probabilistic sensitivity analysis of ROTARIX (with Gavi support) and ROTASIIL (without Gavi support) over the period 2021–2030.

With Gavi support, the only non-dominated product is ROTARIX, and there is a 98 % probability it will be cost-effective at a WTP threshold set at 0.5x GDP per capita. Without Gavi support, the only non-dominated product is ROTASIIL, and there is 30 % probability that it will be cost-effective at the same threshold (Fig. 3).

### 3.3 Scenario analysis

As shown in the Supplementary file - Fig. 1, all three vaccines were cost-effective at a threshold of 0.5x GDP per capita (US$224) in most of the scenarios evaluated when compared to no vaccination. At the Gavi-subsidized price, ROTARIX has the most favourable cost per DALY averted (US$102). At the vaccine price without Gavi support, ROTASIIL was the most cost-effective vaccine at US$259 per DALY averted. The most influential parameters identified in deterministic scenario analyses were the burden of disease, the incremental delivery cost, and the vaccine price.

When we estimate the cost-effectiveness of ROTAVAC, ROTASIIL, and ROTARIX as if they have the same health impact, the rank order did not change, ROTARIX still represented the best option with Gavi support. When doing the same exercise using vaccine price without Gavi support, we observed that ROTASIIL was the best option.

## 4 Discussion

We estimate that ROTARIX vaccination may have prevented over 4,500 deaths between 2016 and 2020. This more than 40 % reduction in deaths is broadly consistent with the real-world impact of ROTARIX observed in Mozambique. When comparing the pre-vaccine and post-vaccine period, de Deus *et al.* [11] found that the vaccine halved the RV-positive proportion among diarrhea hospital admissions. We also found similar reductions in severe RV disease cases, clinic visits, and hospitalizations.

Compared to no RV vaccine, the use of ROTARIX with Gavi support in Mozambique’s immunization program from 2016 to 2020 was cost-effective (US$70 per DALY averted). Even in scenarios with the least favourable incremental delivery cost and the lowest vaccine efficacy (49.9 %), the cost-effectiveness results were still favourable (US$199 and US$100 per DALY averted, respectively). ROTARIX would continue to be cost-effective from a government perspective if Gavi support were to continue throughout the period 2021–2030. Our modelling found that ROTARIX was the most cost-effective option despite averting fewer RVGE disease events than ROTAVAC and ROTASIIL. When we assumed the same impact for all three vaccines, the cost per DALY averted for ROTARIX was only slightly more favourable (US$89 vs US$102). This difference in health benefits occurs because we assume that all three vaccines confer a similar level of protection at the final dose, and after that, the protection declines over time. Since the last dose of ROTARIX is given earlier, at 4 months of child’s age, the decline in protection begins earlier than with the other vaccines, which have the last dose given at 6 months. This results in lower overall modelled health and economic benefits, which may not reflect real-world differences in vaccine impact [40]. Higher modelled impact for three-dose courses is not based on a head-to-head product comparison. Rather, this finding is the result of a later time point for the final dose for the three-dose products and should be interpreted cautiously.

With Gavi support, vaccination program costs with ROTASIIL and ROTAVAC are higher than with ROTARIX by almost US$1.0 million per year. This is because the former two vaccines are administered in three doses, increasing overall immunization delivery costs by additional US$1.17 per complete vaccine schedule compared to the two-dose ROTARIX. Another important driver of this difference is the wastage rate per dose, which is higher for ROTASIIL and ROTAVAC (9 % and 13 %, respectively) compared to ROTARIX (4 %) [6]. The lower costs calculated for ROTARIX resulted in this vaccine having the most favourable cost-effectiveness ratio (US$102 per DALY averted). This finding is consistent with a previous analysis that aimed to compare the cost-effectiveness of the same vaccines in Bangladesh, Ghana, and Malawi, where ROTARIX was the most cost-effective [44]. Later analysis demonstrates that this finding is sensitive to context and assumptions [45].

In the absence of Gavi support, ROTASIIL was estimated to have the lowest costs and would have the most favourable cost-effectiveness, driven by the lower price of this vaccine. This result differs from the findings in Bangladesh, Ghana, and Malawi, where ROTARIX remained the most cost-effective product even in the absence of Gavi support. This is because the system cost for vaccination was lower than the other vaccines, which made the ratio between costs and gains better for ROTARIX [44]. We found a relatively low probability (around 30 %) that the most favourable product without Gavi support (ROTASIIL) would be cost-effective from a government perspective based on the current WTP threshold set at 0.5x the national GDP per capita. However, when the country reaches Gavi’s fully self-financing phase and utilizes a higher anticipated threshold (e.g., 0.5x US$1,630), the probability for ROTASIIL to be cost-effective is higher than 95 %.

The eligibility threshold for graduation from Gavi support is currently US$1,630 GNI per capita. Similar to other studies [15], [44], [46], a re-evaluation of the cost-effectiveness of RV vaccines and comparison to updated thresholds will be needed before Mozambique starts this process. Budget impact analysis will also be important to show the financial resources that may eventually be required to graduate from Gavi support.

In a situation of scarce resources, as observed in most low-income countries such as Mozambique, ROTARIX represents good value for money for the government while the price is heavily subsidised by Gavi. If Mozambique begins to transition away from Gavi support, then ROTASIIL may be a preferable option, but still may not be as cost-effective from a government perspective at today’s threshold. However, in addition to the cost-effectiveness result, the selection of vaccine product should also consider other aspects as affordability, feasibility, and other country-specific factors [24], [25].

Cost–effectiveness analysis in health is used to compare the costs and outcomes of alternative interventions and is measured by the incremental cost to obtain a unit of health gain [27]. The assessment of whether the intervention is cost-effective is made based on a WTP threshold that represents a good value for money [24]. The WHO Commission on Macroeconomics in Health recommended that the cost-effectiveness thresholds corresponds to up to 3x a country’s per-capita GDP [25]. However, the use of GDP-based thresholds in a decision-making process is less country-specific. Together with uncertainties in the model, their use can lead to the wrong decision on how to choose the intervention and spend health-care resources [24], [25].

Our study had some limitations. First, some of the parameters were based on global estimates or assumptions. To mitigate this aspect, we were able to share these estimates with a national team of experts to ensure consensus around the inputs that were selected, including many context-specific inputs such as vaccine coverage, wastage, system costs, and prices. Second, WHO no longer recommends using generic GDP per capita thresholds to interpret cost-effectiveness results [26]. However, Mozambique has not defined a country-specific WTP threshold, so we used 0.5x GDP per capita to help put our results in context. Third, we have made several assumptions to differentiate the current RV vaccine products on the basis of price, system costs, wastage, and efficacy. These influential parameters are likely to be updated over time, and this analysis should be updated with more relevant data when possible. Fourth, we excluded costs borne by households such as out-of-pocket medical expenses, travel, and lost earnings. However, these costs are likely to be relatively small, and a preliminary analysis with these costs included did not alter the cost-effectiveness results. Finally, UNIVAC is a static model and does not take the herd immunity effect into account. There is currently limited evidence to suggest a substantial herd effects in LMICs; nevertheless, our results should be interpreted as a conservative estimate of the potential health benefits of RV vaccination.

## 5 Conclusion

Vaccination with ROTARIX has already had a substantial public health impact in Mozambique, preventing over 4,500 deaths between 2016 and 2020. With continued Gavi support, ROTARIX remains the most cost-effective product. However, if Mozambique were to fully self-finance the program, ROTASIIL would be preferred but may not be as cost-effective based on current prices and assumptions. The choice of vaccine product should be continually re-evaluated as more evidence emerges about their prices, health system costs, wastage rates, relative health impacts, and also as Mozambique’s Gavi eligibility status and WTP thresholds change.

## Declaration of Competing Interest

The authors declare that they have no known competing financial interests or personal relationships that could have appeared to influence the work reported in this paper.
