# Supplementary material for: Cost-effectiveness of rotavirus vaccination in Mozambique
Source: Vaccine. 2022 Aug 26;40(36):5338–46. doi: 10.1016/j.vaccine.2022.07.044 (PMC9421418; doi:10.1016/j.vaccine.2022.07.044)
Supplement: Supplementary data 1 [file mmc1.pdf]

Supplemental File

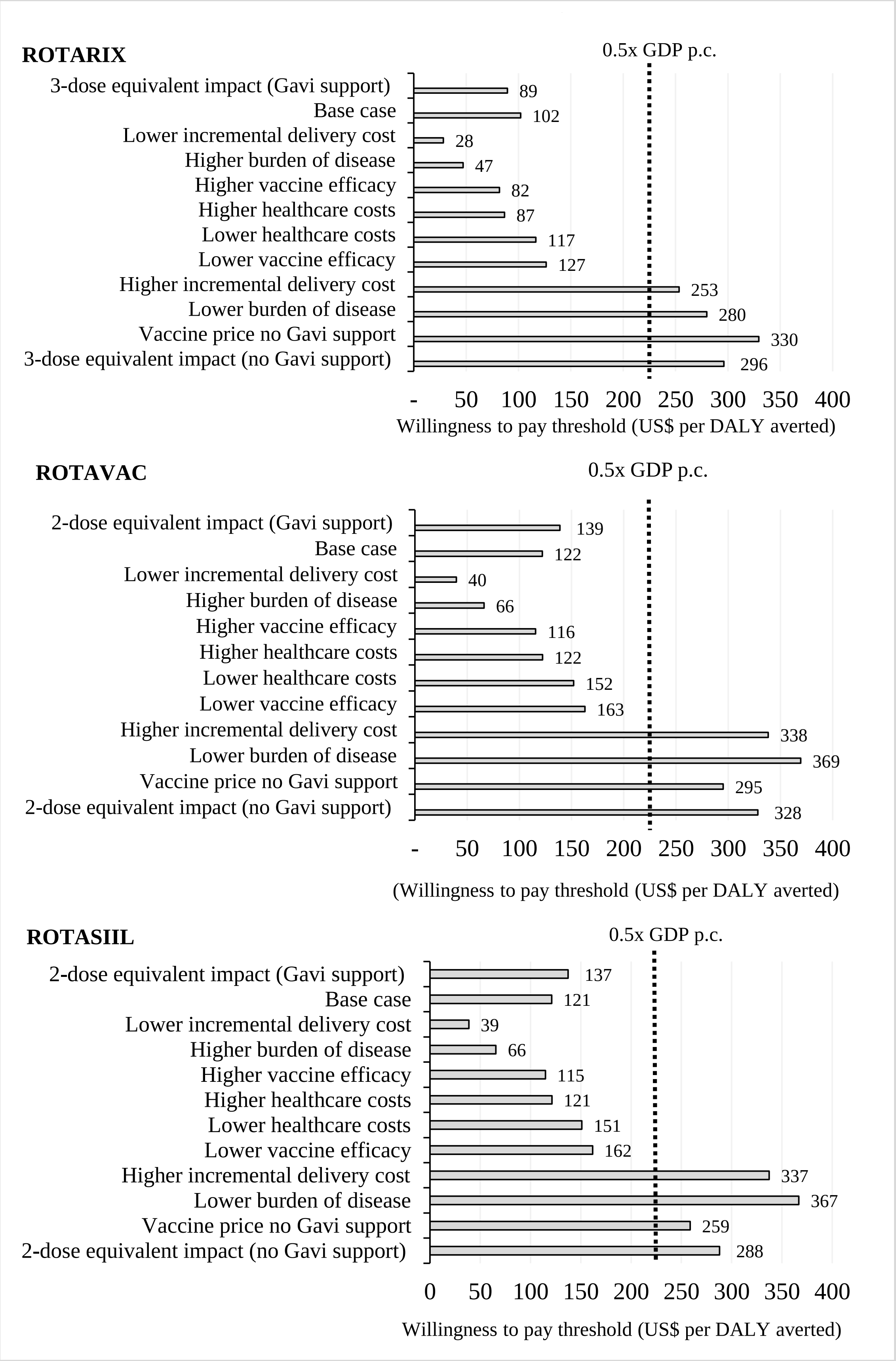

Figure 1. Scenario analysis results, showing incremental cost-effectiveness ratio (US$ per DALY averted) of ROTASIIL, ROTAVAC, and ROTARIX, compared to no vaccination.
Legend: GDP p.c.: gross domestic product per capita.
