# Supplementary material for: Cost-effectiveness of rotavirus vaccination in Mozambique
Source: Vaccine. 2022 Aug 26;40(36):5338–46. doi: 10.1016/j.vaccine.2022.07.044 (PMC9421418; doi:10.1016/j.vaccine.2022.07.044)
Supplement: Supplementary data 2 [file mmc2.pdf]

**Supplemental file**

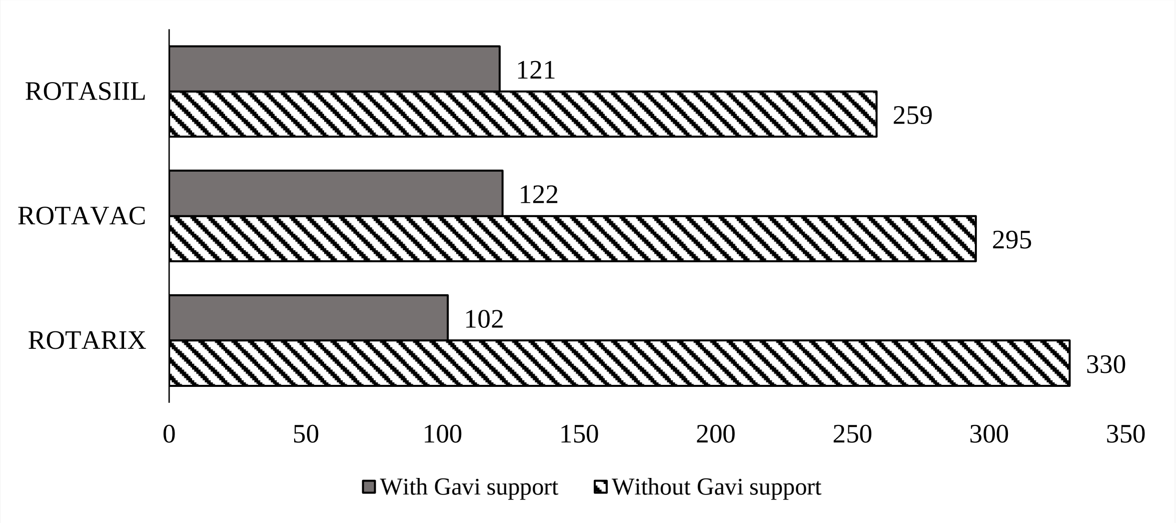


Figure 2. Cost-effectiveness of ROTASIIL, ROTAVAC and ROTARIX vaccines, with and without Gavi support, compared to no vaccination.
